# Molecular Mechanism Analysis of the Activation of Human Olfactory Receptor OR9Q2 by 4-Methylphenol

**DOI:** 10.3390/foods14213738

**Published:** 2025-10-31

**Authors:** Fengge Wen, Mengxue Wang, Lili Zhang, Wen Duan, Baoguo Sun, Jianping Xie, Mingquan Huang, Shihao Sun, Rui Yang, Yuyu Zhang

**Affiliations:** 1 College of Food Science and Engineering, Tianjin University of Science and Technology, Tianjin 300457, China; 13949989546@139.com (F.W.); sherry_1182@163.com (M.W.); yangrui@tust.edu.cn (R.Y.); 2 Beijing Life Science Academy, Beijing 102209, China; xiejp@blsa.com.cn; 3 Key Laboratory of Geriatric Nutrition and Health, Beijing Technology and Business University, Ministry of Education, Beijing 100048, China; zhanglili921116@163.com (L.Z.); 15754367187@163.com (W.D.); sunbg@btbu.edu.cn (B.S.); huangmq@th.btbu.edu.cn (M.H.)

**Keywords:** hOR9Q2, molecular docking, molecular dynamics simulation, site-directed mutagenesis, odorant-receptor interaction

## Abstract

This study employed a combined computational and experimental approach to investigate the molecular recognition mechanism of 4-methylphenol by human olfactory receptor hOR9Q2. The strategy integrated molecular docking using BIOVIA Discovery Studio, structural modeling of hOR9Q2 based on the AlphaFold2-predicted, molecular dynamics simulations with GROMACS software employing the AMBER14SB force field, and systematic site-directed mutagenesis validation. Computational simulations revealed that the binding cavity formed by transmembrane domains TM3, TM5, and TM6 serves as the key interaction region, with van der Waals, hydrophobic, and Pi-sulfur interactions driving stable binding (ΔG = −40.173 ± 0.34 kJ/mol). Functional characterization identified six critical residues (Cys112, Val158, Met207, Phe251, Leu255, and Tyr259) as essential for receptor activation, while mutations at Ile71 and Ala108 resulted in partial functional impairment. This study reveals the structural basis for hOR9Q2’s selective response to 4-methylphenol, while establishing a computational–experimental framework for precisely locating functional sites on olfactory receptors. These findings elucidate the molecular mechanism of odorant recognition and provide insights for developing odorant prediction models and designing specific olfactory receptor modulators.

## 1. Introduction

4-Methylphenol, also known as *p*-cresol, is a naturally occurring organic compound that is found in a variety of food products. It is also detectable in wood, tobacco smoke, crude oil, coal tar, and the urine of animals and humans [1,2]. As an aromatic compound, 4-methylphenol has been identified as an odor-active compound for various food products, including beef, mango, wine, and whiskey [3,4]. It exhibits a distinct phenol-like odor that is often reminiscent of manure typically found in stables, and its low aroma detection threshold (50~100 ppb) further enhances its recognition among various odorants (Fenaroli’s Handbook of Flavor Ingredients). Furthermore, 4-methylphenol plays an important role as a chemical pheromone in information transfer among organisms [5,6]. Studies have demonstrated that 4-methylphenol, in conjunction with other compounds such as indole and 3-methylindole, plays a significant role in producing the off-odors commonly associated with pig intestines [7]. Additionally, the unique odors detected in goat milk and meat have also been traced to the metabolic byproduct 4-methylphenol [8]. Among various phenolic compounds, 4-methylphenol stands out as a characteristic odorant in leather products. Schroepfer et al. reported that 4-methylphenol might be a particularly appealing and preferred odorant in olfactory environments associated with “stables” and “leather”, as well as certain stimulants like “whiskey” and “tobacco” [9].

The olfactory receptors serve as a vital connection between the olfactory system and the spatial contexts that contain odor cues. The fundamental units of this system are the olfactory sensory neuron cilia, which house these receptors and play a crucial role in the overall performance of the olfactory system [10]. These specialized receptor cells are capable of binding to odor molecules through specific olfactory receptor proteins, generating neural signals that convey odor information to the brain. Different olfactory receptor proteins have been shown to recognize a diverse range of odorants, allowing us to perceive a vast array of complex scents [11,12]. In addition to the conserved broad-spectrum olfactory receptors, there is a growing body of evidence suggesting that evolutionarily non-conserved olfactory receptors exhibit a high degree of selectivity for individually characterized key food odorants [13,14,15,16].

Studies have demonstrated that 4-methylphenol specifically activates the olfactory receptor family 9 subfamily Q member 2 (OR9Q2) within the olfactory system [17]. Nevertheless, the molecular mechanism by which 4-methylphenol activates the olfactory receptor OR9Q2 is unclear. With rapid advancements in artificial intelligence and computational technologies, innovative methodologies such as molecular docking and molecular dynamics simulation are emerging as powerful tools for understanding the intricate mechanisms of interaction between olfactory receptors and their corresponding ligands [18,19]. The development of AlphaFold2 represented a pivotal moment, showcasing extraordinary accuracy in predicting the three-dimensional structures of a vast array of proteins [20]. This capability allows researchers to move beyond traditional homology modeling and directly employ models from the AlphaFold2 database as reliable starting points for investigation [21]. This progress is particularly relevant in olfactory research, as demonstrated by Kaneshiro et al., who utilized AlphaFold2 predicted structures of 409 human olfactory receptors in molecular docking simulations to study odor similarity [21]. The availability of such structural data enhances the utility of computational methods like molecular docking, a critical tool for predicting ligand–receptor binding modes and affinities, thereby streamlining the processes of ligand selection and optimization [22]. For instance, Zhu et al. utilized molecular docking and molecular dynamics simulations to investigate the interactions of key chiral lactone compounds (γ-octalactone and γ-undecalactone) in Longjing tea with the olfactory receptor OR1A1, revealing the molecular basis of their odor recognition mechanisms [23]. Mei et al. utilized molecular docking techniques to delve into the binding mechanisms of key odorants found in tea with olfactory receptors and found that compounds such as nonanal and methyl salicylate produce distinct odors by activating specific olfactory receptors [24]. Similarly, Chaohui et al. harnessed this technology to investigate the mechanisms by which novel umami peptides enhance the flavor profiles of Yanjin black bone chicken meat, further illustrating the versatility and applicability of molecular docking in flavor research [25]. Although molecular docking is the most commonly used tool for predicting ligand–receptor binding modes, its scoring functions are approximate and often fail to accurately account for solvation effects and true binding free energy, making it more suitable for rapid initial screening [26]. In contrast, molecular dynamics simulations can capture induced-fit effects and large-scale conformational changes, while methods such as Molecular Mechanics Poisson–Boltzmann Surface Area or Generalized Born Surface Area (MM-PBSA/GBSA) provide more accurate binding free energies [27]. Moreover, trajectory analysis offers dynamic metrics such as Root Mean Square Deviation (RMSD) and hydrogen bond lifetime, which serve as independent validation of the molecular docking results and significantly enhance the reliability of the predictions [27].

Validating computational simulation results through cellular experiments is a crucial part of revealing the intricate mechanisms underlying receptor–ligand interactions. Site-directed mutagenesis has proven to be exceptionally beneficial in uncovering the essential active sites of receptor proteins [28,29,30]. This approach enables the precise introduction of targeted mutations within amino acid sequences, modifying the corresponding amino acid sequence and the overall protein structure. This in turn helps researchers to identify the pivotal active sites of receptor proteins [31,32]. Li et al. successfully identified the critical residues contributing to the indole-driven activation of associated receptors through the application of molecular docking and mutagenesis techniques [7]. Taken together, the synergistic integration of homology modeling, molecular docking, and cellular experimental methodologies is an effective way to illuminate the interaction mechanisms between olfactory receptors and odorants.

This study aims to elucidate the molecular mechanism of 4-methylphenol binding to human olfactory receptor OR9Q2 through a computational simulation techniques and cell experimental approach. The specific objectives were:

- To characterize the binding mode and identify key residues involved in 4-methylphenol recognition through molecular docking.
- To evaluate the binding stability and quantify binding affinity using molecular dynamics simulations and MM-PBSA analysis.
- To evaluate the activation of 4-methylphenol on hOR9Q2 by constructing hOR9Q2-expressing HEK293 cells.
- To confirm the functional significance of predicted residues through site-directed mutagenesis.

This work will contribute to a deeper understanding of the detailed molecular mechanism by which 4-methylphenol activates hOR9Q2, providing innovative perspectives and methodologies for the perception mechanism analysis of odorants.

## 2. Materials and Methods

### 2.1. Chemicals

The chemicals used in this study were obtained as follows: 4-Methylphenol with a purity of ≥99.7% was purchased from the Aladdin website. Dulbecco’s Modified Eagle Medium (DMEM), Opti-MEM medium, and Hank’s solution were purchased from Gibco (Grand Island, NY, USA). The PCI-Neo vector and the cAMP-Glo™ Assay were procured from Promega (Woods Hollow Road, Madison, WI, USA). Fetal Bovine Serum (FBS) was obtained from PAN-Biotech (Aidenbach, Germany). The plasmid mini-prep kit was purchased from Omega (Norcross, GA, USA). Linear polyethylenimine with a molecular weight of 25,000 was purchased from Polyscience (Chicago, IL, USA). Human embryonic kidney (HEK) 293 cells were obtained from the American Type Culture Collection (ATCC) (Manassas, VA, USA). The high-purity plasmid midiprep kit (DP107) was purchased from Tiangen Biotech (Beijing, China). Competent DH5α cells and the Mut Express^®^ IIFast Mutagenesis Kit V2 were obtained from Vazyme Biotech (Nanjing, China). Finally, ultrapure water was obtained from Watsons Food & Beverage Co., Ltd. (Guangzhou, China).

### 2.2. Determination of Odor Threshold

A total of 15 sensory assessors (8 females and 7 males, with a mean age of 24) were recruited from the Key Laboratory of Flavor Science of China General Chamber of Commerce (Beijing, China) in accordance with ISO 8586:2023 [33]. All assessors met the standard’s criteria for experienced sensory assessors, having (1) completed over six months of formal training in odor identification and intensity scaling, (2) demonstrated consistent performance in standardized tests for odor recognition and quantification, and (3) accumulated substantial practical experience in the sensory profiling of food-related odorants through regular participation in sensory evaluation sessions. Prior to the initiation of sensory experiments, an application was submitted to and approved by the Ethics Committee of Beijing Technology and Business University (BTBU202333, Beijing, China, March 2023–March 2026. All participants were non-smokers without rhinitis, participated voluntarily, and reserved the right to withdraw from the study at any point. After comprehensively understanding the research requirements and associated risks, the participants signed an informed consent form (Supplementary Materials).

The panelists were trained for one week (2 h per day) prior to undertaking the three-alternative forced choice (3-AFC) analysis. Initially, a higher concentration of the compound was selected, followed by two or more stimulus concentrations, and the panelists were tasked with ranking them. The evaluators’ performance reached stability after 3 to 5 assessments. Once the preference detection threshold range for the panelists was determined, the 3-AFC method was implemented for testing.

The detection threshold of the compounds was carried out via the 3-AFC method according to the general guidance for odor detection thresholds in ISO 13301:2018 [34]. Among the three test samples, only one contained the odorant and the other two acted as blank controls. The initial concentration of the 4-methylphenol solution was set at 0.2 µL/L with a dilution factor of 2, resulting in a total of 10 dilution gradients in this work. The samples were presented in descending order of odorant concentration during the test. The participants were instructed to sniff three samples per group and then identify the one that differed from the other two. If the selected sample contained the odorant, the test would proceed to the next group; otherwise, the test would be terminated. Finally, the logistic function was fitted to the concentration of the compound and the corresponding accuracy using Origin 2021. A concentration corresponding to an accuracy of 0.5 was considered the detection threshold concentration.

### 2.3. Structure Model of hOR9Q2 and 4-Methylphenol

The three-dimensional structure of the hOR9Q2 was obtained from the AlphaFold2 Protein Structure Database (https://alphafold.com/ (accessed on 20 June 2024)). This predicted model was selected for its high predicted Local Distance Difference Test (pLDDT) score (pLDDT = 85.19), with the majority of residues, particularly within the transmembrane and putative binding pocket regions, falling within the high-confidence range (pLDDT > 70). The amino acid sequences were retrieved from the National Center for Biotechnology Information (NCBI) (https://www.ncbi.nlm.nih.gov/ (accessed on 25 June 2024)). The relevant NCBI reference sequences are as follows: Homo sapiens, NP_001005283.1; Pan troglodytes, XP_054518272.1; Macaca mulatta, XP_002808115.2; and Mus musculus, NP_666952.1. The three-dimensional structure of the ligand, 4-methylphenol, was downloaded from the PubChem database (https://pubchem.ncbi.nlm.nih.gov/ (accessed on 20 June 2024)).

### 2.4. Molecular Docking

The PlayMolecule platform (https://open.playmolecule.org/ (accessed on 20 June 2024)) was employed to preprocess olfactory receptor proteins and forecast their binding pockets, whose dimensions were utilized to establish the docking box for molecular docking procedures. The protein structure was prepared for docking using the standard “Prepare Protein” protocol in BIOVIA Discovery Studio 2021, which included the addition of hydrogen atoms and charge assignment. The three-dimensional structures of the olfactory receptor hOR9Q2 and 4-methylphenol were imported into BIOVIA Discovery Studio 2021, and then molecular docking was performed using the CDOCKER module in the software. Docking calculations generated 11 poses, which were ranked based on their CDOCKER Interaction Energy. The conformation with the most favorable interaction energy, which also exhibited key interactions with the binding site residues, was selected as the optimal binding mode for further analysis. The two-dimensional interaction maps were created using Discovery Studio and PyMOL 3.1.0 for the visualization of the binding sites and binding forces.

### 2.5. Molecular Dynamics (MD) Simulations

The MD simulations were performed using GROMACS 2022 software. The olfactory receptor hOR9Q2 was parameterized using the AMBER14SB force field with a TIP3P water model [35], while the ligand 4-methylphenol was utilized using the GAFF force field. Under periodic boundary conditions, energy minimization was conducted first, followed by equilibration phases of 100 ps in the NVT ensemble at 298 K and NPT ensemble at 1 bar. Subsequently, a 100 ns molecular dynamics simulation was performed. During the simulation, the LINCS algorithm constrained hydrogen bonds, with a time step of 2 fs. Electrostatic interactions were calculated using the Particle Mesh Ewald (PME) method with a cutoff radius of 1.2 nm.

### 2.6. Calculation of Binding Free Energy Change

Considering solvation energy, the binding free energy change (ΔG_bind_) was computed by integrating the root mean square deviation (RMSD), radius of gyration (Rg), interatomic distances, buried solvent-accessible surface area (SASA), and interaction energies from trajectories of stable complexes. The MM-PBSA method was employed to derive the energetic contributions associated with ligand–receptor binding. ΔG_bind_ represents the Gibbs free energy variation during the formation of the ligand–receptor complex under constant temperature and pressure conditions. A more negative ΔG_bind_ indicates a higher spontaneity and affinity of binding. It can be expressed as (1) [36,37].

ΔG_bind_ = ΔG_gas_ + ΔG_sol_ − TΔS (1)

Gas-Phase Binding Free Energy Change (ΔG_gas_) refers to the change in free energy that occurs when a ligand binds to a receptor in a vacuum environment (without solvents). Essentially, it is the net sum of all molecular mechanical interactions between the two, which can be expressed by (2) [38]. Among them, the Energy change of the bonding term (ΔE_bonded_) includes energy changes such as bond length stretching (chemical bonds), bond angle bending, and dihdral angle rotation (torsion), but in protein-small molecule binding calculations, ΔE_bonded_ is usually ignored or considered approximately zero. The Energy change of non-bonded terms (ΔE_nonbonded_) originates from non-covalent interactions, which are the main driving forces for molecular binding, including Electrostatic Energy Change (ΔE_ele_) and van der Waals Energy Change (ΔE_vdW_).

ΔG_gas_ ≈ ΔE_MM_ = ΔE_bonded_ + ΔE_nonbonded_ = ΔE_bonded_ + (ΔE_ele_ + ΔE_vdW_) (2)

The polar solvation free energy (ΔG_sol_) refers to the net change in free energy resulting from the reorganization of the solvent environment during the binding process. It can be expressed by (3) [39]. Here, the polar solvation energy change (ΔG_PB_) is calculated using the Poisson-Boltzmann equation (PB). The non-polar solvation energy change (ΔG_SA_) mainly results from the reduction in the solvent accessible surface area (SASA) during the binding process, driven by the hydrophobic effect. It is usually proportional to the change in SASA and is one of the main driving forces for binding.

ΔG_sol_ = ΔG_PB_ + ΔG_SA_ (3)

−TΔS is the product of temperature (T) and the change in entropy (ΔS) of the system during the binding process. Due to the significant error in the calculation of −TΔS, this term is often not included in the comparison of binding energies.

### 2.7. Molecular Cloning of hOR9Q2

The cDNA sequence of hOR9Q2 was synthesized (NCBI reference sequence: NM_001005288.3), and the target gene was cloned into the PCI-Neo vector via the recombinant method (cloning site: For ECORI 5 ‘3’ for NotI). After cloning, the recombinant result was transformed into DH5α competent cells, and positive clones were chosen for sequencing. The monoclonal bacteria in the plates were amplified, and the plasmids were extracted when the sequencing results matched the sequences on the PubMed website (https://pubmed.ncbi.nlm.nih.gov/ (accessed on 10 July 2024)).

### 2.8. PCR-Based Site-Directed Mutagenesis

The hOR9Q2 variants were constructed using Mut Express^®^ II Fast Mutagenesis Kit V2 (Vazyme Biotech Co., Ltd, Nanjing, China), a targeted mutagenesis system based on Clon Express rapid cloning technology. The target gene was amplified using Phanta Max Super-Fidelity DNA Polymerase (Vazyme Biotech Co., Ltd, Nanjing, China), and the amplified product was transformed into DH5α competent cells and incubated in inverted plates at 37 °C for 14 h. Two positive clones were selected for sequencing at each mutation site.

The 50 μL target gene amplification system consisted of 2× Max Buffer (25 μL), dNTP Mix (10 mM, 1 μL), template DNA (1 μL), forward primer (10 μM, 1 μL), reverse primer (10 μM, 1 μL), Phanta Max Super-Fidelity DNA Polymerase (1 μL), and ddH_2_O (18 μL). The PCR amplification of the target gene was performed under the following conditions: Initially, the reaction was conducted at 95 °C for 30 s to achieve pre-mutation. Thirty cycles of the reaction were performed at 95 °C (15 s), 65 °C (15 s), and 72 °C (3.5 min) to amplify the target DNA. Finally, the reaction was performed at 72 °C for 5 min to ensure complete extension.

### 2.9. Cell Culture and Transfection

HEK 293 cells were used as the cell lines for the functional expression of recombinant olfactory receptors. The cells were cultured in DMEM supplemented with 10% FBS at 37 °C in an incubator with 5% CO_2_. The cultured cells were grown in logarithmic phase and detached with 0.25% trypsin when they reached 90% confluency. The detached cells were then resuspended in complete medium for plating or passaging.

Then, 1 mL of medium containing HEK 293 cells at a density of 1 × 10^5^/mL was added to a 12-well plate and incubated at 37 °C in an incubator with 5% CO_2_ for 20 h before transfection. PEI transfection reagents were used for gene delivery. In an EP tube, 100 μL of Opti-MEM solution was prepared, to which 1 μg of plasmid OR9Q2 (control plasmid pCl-eo), 0.5 μg Gαolf, 0.5 μg RTP1S, and 1 μg of cAMP-luciferase pGloSensor-22F plasmids were added, followed by 12 μL PEI. The mixture was vortexed and allowed to stand at room temperature for 20 min. The resulting solution was carefully added to the wells of a plate and cultured for an additional 24 h for further experiments.

### 2.10. Luminescence Assay

Cells cultured for 24 h post-transfection were digested with 0.25% trypsin-EDTA. The digestion was terminated with DMEM containing 10% FBS, and the supernatant was removed via centrifugation at 250 g. The cells were then resuspended in a solution containing 3% *v*/*v* GloSensor cAMPs and 10% FBS, and the cell density was adjusted to 1 × 10^5^/mL. Subsequently, 95 μL of the cell suspension was added to each well of the plate. After 2 h of incubation at room temperature in the dark, the odorants were added to each well and mixed gently for 5 s. Luminescence was then measured using a microplate reader (Tecan Spark, Männedorf, Switzerland).

### 2.11. Data Analysis

Microsoft Office Excel was used for data organization and basic statistical calculations; IBM SPSS Statistics 27 was employed for normality testing (Shapiro–Wilk test), homogeneity of variances testing (Levene’s test), and one-way ANOVA with post hoc Duncan’s test; while Origin 2021 and GraphPad Prism 9.5.0 were utilized for nonlinear regression analysis and graph generation.

## 3. Results and Discussion

### 3.1. Odor Characteristics and Thresholds of 4-Methylphenol

**Figure 1:**
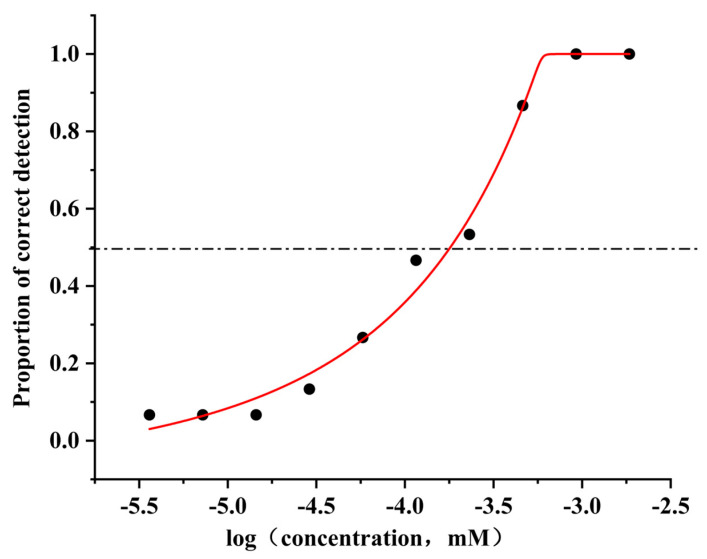
Odor detection threshold of 4-methylphenol.

Among the diverse array of food-flavoring agents, 4-methylphenol stands out due to its unique and distinctive odor characteristics. The diluted solution of 4-methylphenol (0.2 µL/L) has characteristic odors that can be likened to fertilizers, stables, tobacco, and leather, which is consistent with its descriptions in the literature [17,40]. The results of the odor detection threshold evaluation of 4-methylphenol by 15 panelists are shown in Figure 1. It can be concluded that its detection threshold is 0.15 μM when the proportion of correct detections is 0.5. A review of the relevant literature indicates that the detection threshold of 4-methylphenol in water ranges from 0.025 μM to 1.85 μM [41,42]. The detection threshold determined in this work is consistent with the ranges reported in the literature, reinforcing the validity of these findings.

### 3.2. Analysis of Molecular Docking Results

**Figure 2:**
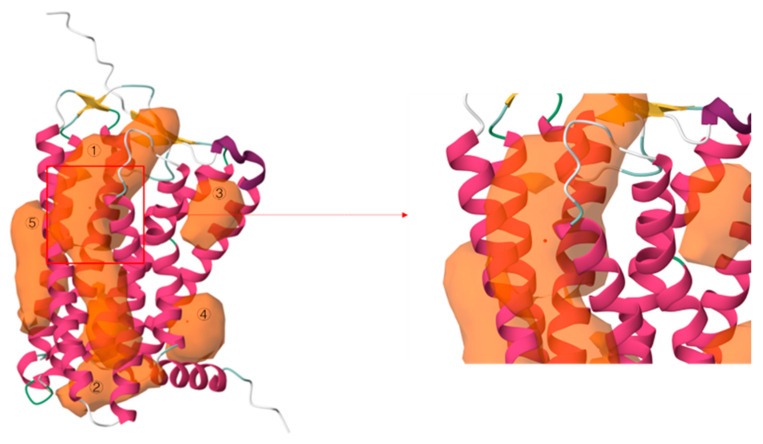
Binding pockets predicted in hOR9Q2.

hOR9Q2 is an olfactory receptor protein with 314 amino acid residues and is characterized by the presence of seven transmembrane (TM) helical domains. As shown in Figure 2, five binding pockets were predicted using the computational software, which are located in (1) TM3, TM4, TM5, and TM6; (2) TM2, TM3, TM6, and TM7; (3) TM1 and TM2; (4) TM1 and TM7; and (5) TM4. It is noteworthy that pocket 1 achieved the highest prediction score among the identified pockets. As a result, pocket 1 was used as the docking box for molecular docking in this work.

**Figure 3:**
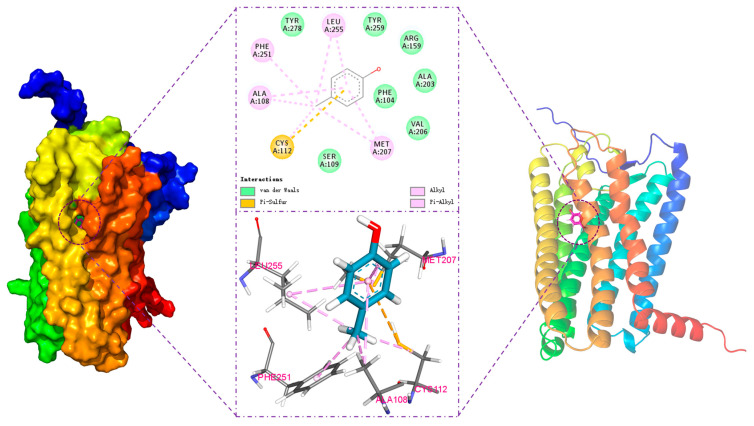
The 2D and 3D interaction diagrams of the optimal conformation for molecular docking. The upper panel presents the two-dimensional (2D) ligand-protein interaction diagram. The lower panel displays the corresponding three-dimensional (3D) binding mode.

The interaction between the odorant and olfactory receptor was analyzed in the designated docking box, and the results are shown in Figure 3. This figure illustrates the optimal binding conformation of 4-methylphenol in hOR9Q2 and highlights the potential existence of five key binding sites in the 4-methylphenol-hOR9Q2 complex, with the following positions: (1) TM3: Ala108, Cys112; (2) TM5: Met207; and (3) TM6: Phe251, Leu255.

Hydrophobic forces are one of the main drivers of protein–ligand binding, in which hydrophobic residues repel water and other polar groups, leading to a net attraction of the ligand’s non-polar groups [43]. Typically, this binding process is accompanied by notable changes in entropy and enthalpy, which suggests that the binding process is primarily entropy-driven [43,44]. The hydrophobic interactions between 4-methylphenol and the amino acid residues Ala108, Cys112, Met207, Phe251, and Leu255 were particularly pronounced in this work. As shown in Table 1 and Figure 3, the alkyl side chains of Ala108, Cys112, Met207, and Leu255 engage in alkyl-hydrophobic interactions with the carbon atoms of 4-methylphenol, with measured distances of 3.9 Å, 4.3 Å, 5.2 Å, and 4.9 Å, respectively. Furthermore, there are Pi-alkyl-hydrophobic interactions between Ala108, Met207, Phe251, Leu255 and the ligand, with distances of 4.5 Å, 4.8 Å, 4.6 Å, and 5.8 Å, respectively. Among these three residues, Phe251 is notable for containing an aromatic ring, a structural feature that is widely acknowledged for its critical role in mediating protein–ligand interactions. Aromatic amino acid residues, such as phenylalanine, frequently participate in binding with ligands that also contain aromatic properties, resulting in Pi-Pi interaction forces. These aromatic residues possess unique electronic characteristics and spatial conformations that enhance their polarizability and quadrupole moments, which contribute to the establishment of preferred geometric arrangements during interactions, ultimately influencing the efficacy and specificity of binding events in biochemical systems [45,46]. In the present work, Phe251 is predicted to establish Pi-alkyl-hydrophobic interactions with the aromatic ring rather than the more frequently observed Pi-Pi interaction. The formation of Pi-Pi stacking interactions is conditional according to McGaughey et al.; therefore, it is inferred that the aromatic ring between the residue Phe251 and 4-methylphenol in the present work does not meet the requirements for the initiation of Pi-Pi interaction [47].

From Figure 3, the residue Cys112 forms a Pi-sulfur bond with the aromatic ring of 4-methylphenol at a distance of 5.0 Å. The Pi-sulfur interaction is ubiquitous in biochemistry and commonly observed between sulfur-containing amino acid residues (e.g., cysteine) and compounds containing aromatic rings. This interaction plays a significant role in enhancing the binding affinity of small molecules to proteins, as well as influencing the conformational stability of these molecules. Studies have analyzed the models of cysteine–aromatic compound interactions and indicated that the lowest-binding-energy conditions for the dehydrogenated configuration of H_2_S-benzene occurred at R = 5.5 Å and θ = 90° [48], which are similar to the results of the present work.

### 3.3. Stability Analysis of the hOR9Q2-4-Methylphenol Complex

**Figure 4:**
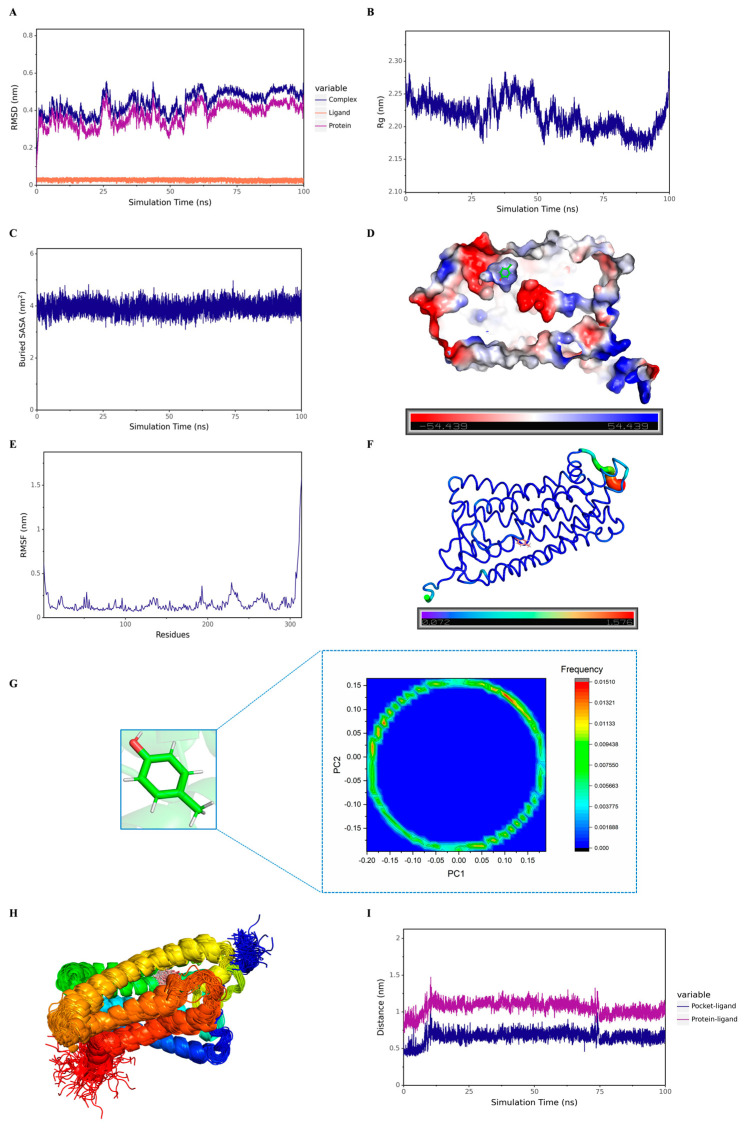
Stability analysis results of the hOR9Q2-4-methylphenol complex. (**A**): The RMSD of the complex, proteins and small molecule ligands. (**B**): The Rg of the complex, and the ordinate is Rg (nm), and the abscissa is time (ns). (**C**): The buried surface area of interaction between small molecules and proteins (Buried SASA), where the ordinate is SASA (nm^2^) and the abscissa is time (ns). (**D**): The surface electrostatic potential of small molecule binding proteins (kcal/mol); (**E**): The RMSF of the protein in the complex, where the ordinate is RMSF (nm) and the abscissa is residue; (**F**): B-Factor (nm). (**G**): Protein binding state structure and PCA. (**H**): Simulated conformation overlaying. (**I**): The distance (nm) between the protein and the binding site of small molecules (Dock site–ligand), where and the abscissa is time (ns).

A multi-dimensional analysis of the binding trajectories obtained from MD simulations was conducted to explore the interaction mechanism between hOR9Q2 and 4-methylphenol as well as the stability of the complex deeply. Firstly, by calculating the root mean square deviation of the complex structure relative to the initial conformation, the overall stability of the system was evaluated [49]. As shown in Figure 4A, the RMSD value of the complex gradually stabilized as the simulation progressed, indicating that the complex structure had reached an equilibrium state and the conformation became stable. This overall stability was further supported by the analysis of the Rg. Rg serves as a crucial parameter for assessing the overall structural compactness of protein-small molecule complexes, while a smaller Rg value indicates a more compact molecular structure [49]. As shown in Figure 4B, the Rg of the complex remained essentially stable throughout the simulation indicating that the binding of the hOR9Q2 and 4-methylphenol maintained stability.

The Buried Solvent Accessible Surface Area (Buried SASA) was analyzed to assess the characteristics of the binding interface, which reflects the surface area buried by the binding interaction and no longer exposed to the solvent [50]. A higher Buried SASA value indicates stronger intermolecular interactions and a larger contact area. As shown in Figure 4C, the value of Buried SASA gradually stabilized during the simulation, indicating that the contact area and binding between the hOR9Q2 and 4-methylphenol gradually stabilize. Additionally, the analysis of the surface electrostatic potential of the binding site (Figure 4D) showed that the surface of the protein groove where 4-methylphenol binds was mainly positively charged, and this charge distribution characteristic facilitated the interaction of small molecules with the protein through electrostatic attraction and hydrogen bonds, thereby stabilizing their binding.

Root mean square fluctuation (RMSF) was calculated to quantify the flexibility of the protein amino acid residues during the MD simulation [51]. As shown in Figure 4E,F, mapping the RMSF values onto the protein structure (displayed in B-Factor form) revealed that the residues around the 4-methylphenol binding pocket generally exhibited lower flexibility, indicating more stable interactions between hOR9Q2 and 4-methylphenol.

Finally, the conformational behavior of the small molecule itself was focused on, with principal component analysis (PCA) used to extract its main motion patterns [52]. The PCA results (Figure 4G) showed that the movement of the 4-methylphenol was concentrated in the projection space, indicating that the conformational fluctuation of the molecule was limited in the binding state [52]. This conclusion was directly verified by the direct superposition of all the 4-methylphenol conformations in the simulation trajectory (Figure 4H), and the high degree of superposition confirmed that 4-methylphenol was stably bound to the protein pocket. At the same time, the distance changes between the 4-methylphenol and the hOR9Q2 core was monitored. From Figure 4I, this distance fluctuated briefly in the early stage of the simulation and reached a stable state, further confirming from the geometric space that the small molecule was stably “locked” at the target binding site.

In summary, multiple evidence chains including RMSD, Rg, Buried SASA, surface electrostatic potential, RMSF, PCA, distance analysis, and conformational superposition indicated that a structurally stable and tightly interacting binding mode formed between the hOR9Q2-4-methylphenol complex during the MD simulation, providing a solid foundation for their physiological function.

### 3.4. Analysis of Hydrogen Bond Interaction Between 4-Methylphenol and hOR9Q2

**Figure 5:**
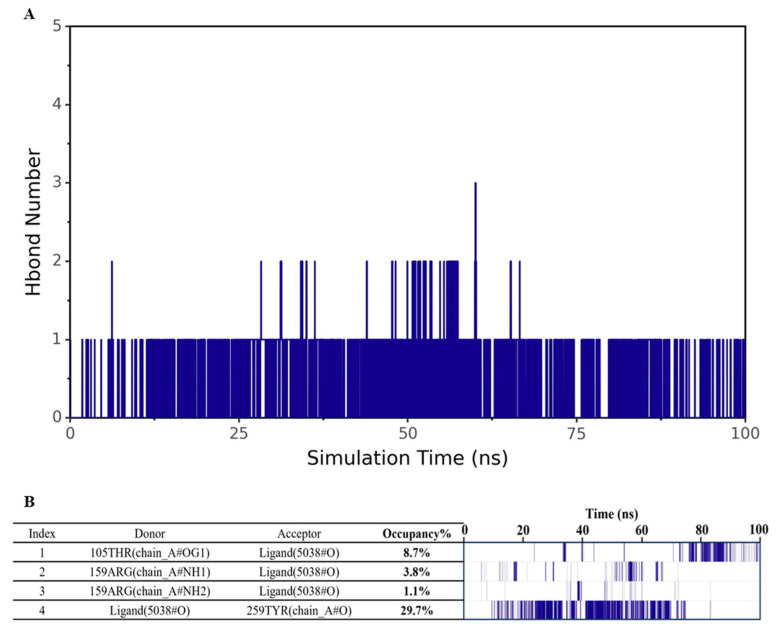
Hydrogen bond interaction analysis diagram. (**A**): The number of hydrogen bonds formed during the entire simulation process. (**B**): The hydrogen bond frequency formed throughout the simulation process.

Hydrogen bonds, as a key interaction force in protein-ligand binding, are closely related to electrostatic interactions and can be used as an important indicator to evaluate the strength of electrostatic complementarity at the binding interface [44]. To systematically assess the hydrogen bonding characteristics between 4-methylphenol and hOR9Q2, the hydrogen bond quantity and formation frequency were analyzed separately. As shown in Figure 5A, the number of hydrogen bonds formed between 4-methylphenol and hOR9Q2 throughout the MD simulation was relatively small, fluctuating mainly between 0 and 1, which indicated that hydrogen bonds are not the dominant force in the binding of this complex. To further explore the stability and contribution of hydrogen bonds, a hydrogen bond frequency analysis was conducted. As shown in Figure 5B, although hydrogen bond interactions can be detected, their formation frequency is generally low, and the corresponding hydrogen bond occupancy is also relatively limited. No stable and frequent hydrogen bonds were found with any specific residue. For example, Tyr259 exhibited a hydrogen bond occupancy of only 29.7%, indicating that this interaction was relatively unstable and insufficient to play a dominant role in binding. Although this residue possessed the potential to form hydrogen bonds, its weak electrostatic contribution (−0.204 kcal/mol) failed to compensate for the high desolvation penalty (+5.024 kcal/mol). The net binding free energy contribution of residue Tyr259 (−5.263 ± 0.332 kcal/mol) originated primarily from strong van der Waals interactions (−9.477 kcal/mol), which not only offset the desolvation cost but also served as the primary driving force for binding—a conclusion that was corroborated by the results presented in Figure 3.

### 3.5. Thermodynamic Study of 4-Methylphenol and hOR9Q2

As shown in Table 2, the total binding free energy of 4-methylphenol and hOR9Q2 was determined to be −40.173 ± 0.340 kcal/mol, indicating a strong spontaneous binding affinity with the receptor. Decomposition of this energy revealed that van der Waals interactions (−81.451 ± 0.351 kcal/mol) served as the dominant driving force, whereas electrostatic contributions (−4.850 ± 0.091 kcal/mol) provided only a modest favorable effect. A pronounced polar solvation penalty (+56.924 ± 0.205 kcal/mol) was observed, reflecting the substantial energetic cost incurred upon desolvation of polar groups during binding. In addition, hydrophobic interactions (−10.796 ± 0.061 kcal/mol) further stabilized the complex, yet their magnitude remained secondary to that of the van der Waals term. Consequently, the high affinity of this complex was primarily attributed to the intense van der Waals contacts established at the 4-methylphenol–OR9Q2 interface, while electrostatic and hydrophobic contributions were largely offset by the accompanying desolvation penalty and therefore did not dominate the binding energetics.

### 3.6. Site-Directed Mutagenesis of Active Site Amino Acids

Following the successful expression of hOR9Q2 in a HEK293 model—confirmed by qPCR (transcriptional overexpression), Western blot (protein detection), and functional cAMP response to 4-methylphenol, as shown in Supplementary Material S2—we characterized the concentration–response relationships of its orthologs from other species.

**Figure 6:**
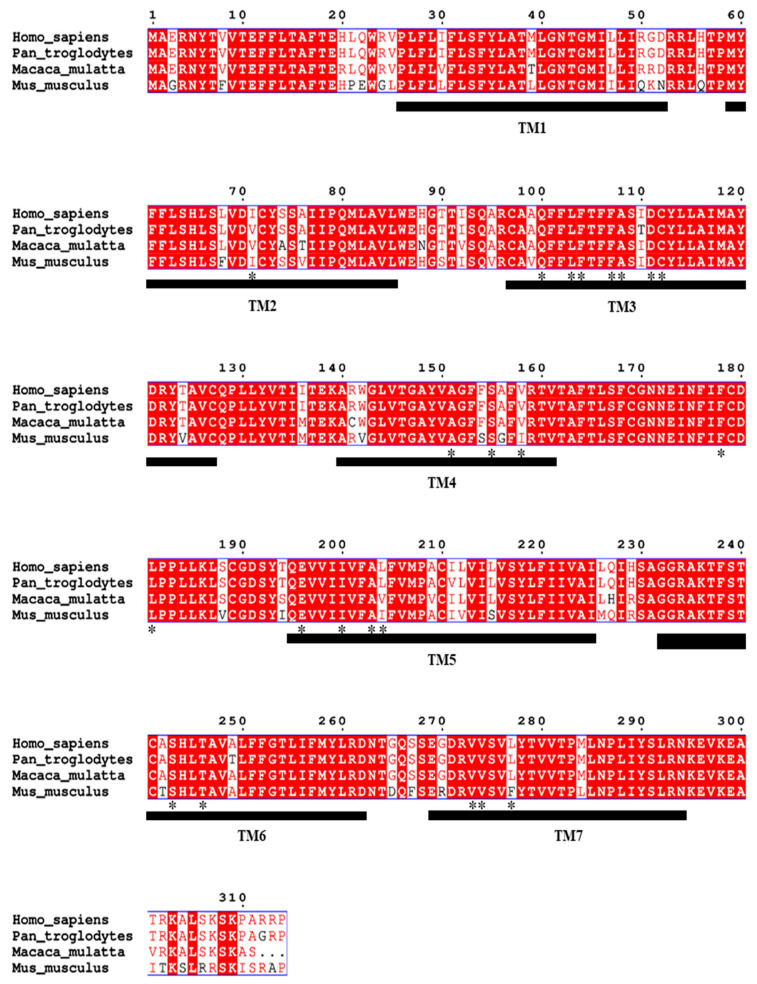
Sequence alignment of OR9Q2 in different species: *Pan troglodytes*, *Mus musculus*, *Macaca mulatta,* and *Homo sapiens*. The TM area is represented by a black horizontal line. The 22 amino acid positions predicted by Man et al. are marked with *, while the different positions of amino acid residues are marked with blank highlights.

The results showed that the amplitude responses of OR9Q2 in chimpanzee (*Pan troglodytes*) and mouse (*Mus musculus*) were significantly lower than in human (*Homo sapiens*) [17]. It is particularly noteworthy that despite the 98% amino acid similarity between chimpanzee and human OR9Q2, the response of chimpanzee OR9Q2 to 4-methylphenol is minimal [17]. It is well known that amino acid residues in the functional regions of proteins tend to be conserved in terms of species evolution, and any alteration of these residues is likely to have a significant impact on the structural stability and function of the protein or even cause protein damage [53]. Twenty-two conserved amino acid positions in the olfactory receptor were predicted by Man et al. [54]. In the present work, sequences of OR9Q2 from chimpanzee, mouse, macaque, and human were aligned, and 4 residues (Ile71, Val158, Leu204, and Leu277) with differences were identified out of the 22 conserved amino acid residues (Figure 6). These 4 residues along with the 6 potential amino acid residues obtained via molecular docking (including residue Tyr259 contributing most to the binding energy), were identified as 10 potential amino acid residues in hOR9Q2, i.e., Ile71, Ala108, Cys112, Val158, Leu204, Met207, Phe251, Leu255, Tyr259, and Leu277. These were selected for site-directed mutagenesis, and the functional expression of the mutated hOR9Q2 was verified.

Relevant information concerning the mutated amino acid residues of hOR9Q2 is detailed in Table 3. Five residues (Cys112, Met207, Phe251, Leu255, and Tyr259) identified via molecular docking were substituted with alanine. Given that alanine possesses a smaller molecular volume relative to other amino acids, this substitution is anticipated to have a minimal effect on the overall protein structure [55]. The replacement of these predicted critical amino acid residues with alanine could result in a reduction or decline in specific protein functionalities, thereby enabling the evaluation of the importance of these amino acid residues in terms of their contribution to protein functionality, active sites, stability, and overall conformation through cellular expression and screening methodologies [30]. Residue Ala108, identified via molecular docking, was mutated to glycine in the present work. Studies have shown that significant disparity in the stability of alanine compared to glycine within the *α*-helices, specifically the mutation of alanine to glycine, alters the solvent-accessible hydrophobic surface area of the protein, consequently influencing its stability [56,57]. Serrano et al. reported that the relative effect of alanine versus glycine on helix stability depends primarily on the position in the helix, with alanine stabilizing it by 0.4 to 2 kcal/mol relative to glycine in the internal position [56]. To avoid the introduction of unnecessary functional groups causing a drastic change in the binding mode, the mutation of alanine to glycine was considered the best option in this study. Four residues (Ile71, Val158, Leu204, and Leu277), which were screened from the conserved amino acid residues of the olfactory receptor, were mutated to the corresponding residues in other species. Specifically, the residues at positions 71, 158, and 277 in the OR9Q2 receptor were mutated to valine, isoleucine, and phenylalanine, respectively. Furthermore, the residue at position 204 was mutated to valine given the higher homology of OR9Q2 in macaque compared to human.

### 3.7. Concentration–Response Relationships of hOR9Q2 and Its Mutants to 4-Methylphenol

**Figure 7:**
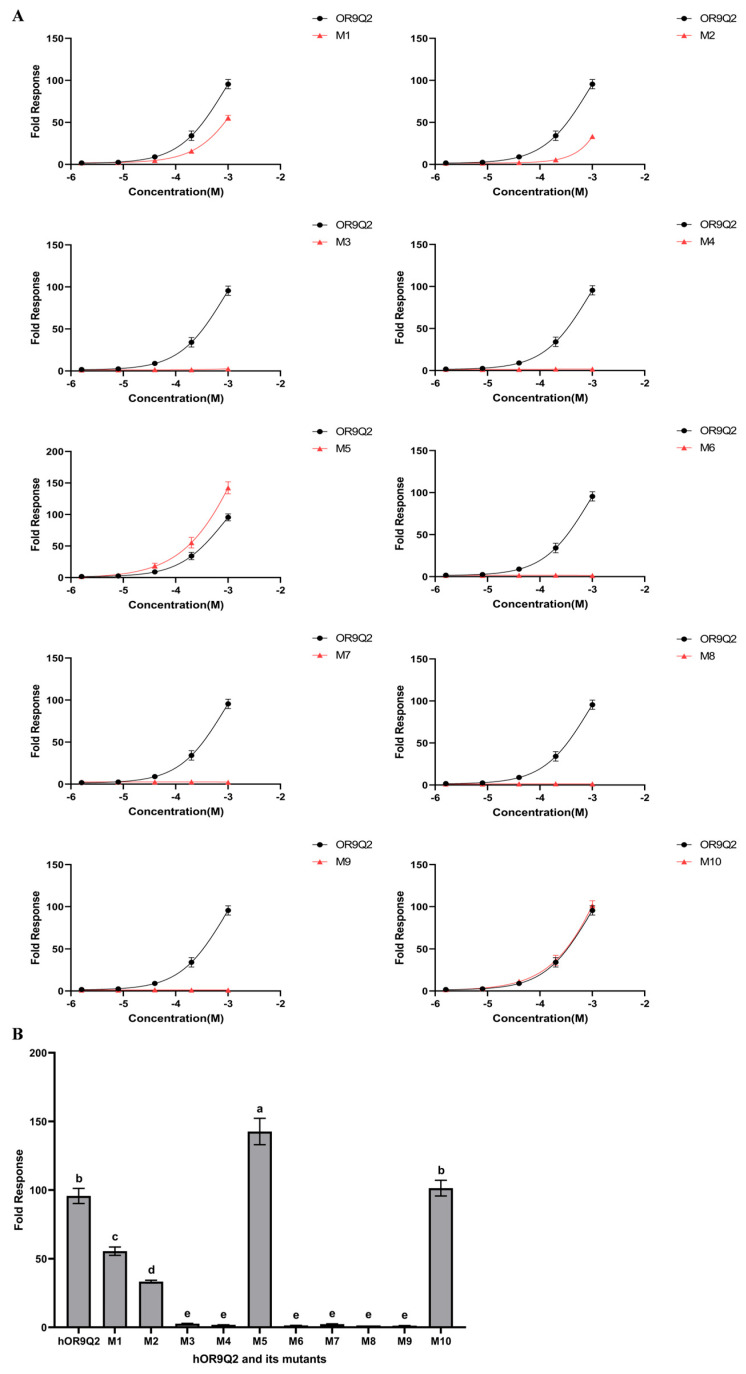
Response of hOR9Q2 to 4-methylphenol after mutation. (**A**) The concentration–response relationships between hOR9Q2 and its mutants to 4-methylphenol, and displayed as mean ± SD (*n* = 3); (**B**) the results of 4-methylphenol (1000 μM) activating the corresponding olfactory receptors. M1–M10 represent mutants of hOR9Q2 in which the 71st, 108th, 112th, 158th, 204th, 207th, 251st, 255th, 259th, and 277th amino acids of the receptor are mutated to Val, Gly, Ala, Ile, Val, Ala, Ala, Ala, Ala and Phe, respectively. Fold response was calculated relative to a control sample containing vehicle alone. Significance analysis was performed on the response results and the different letters (a, b, c, d, and e) indicate significant differences at the *p* < 0.05 level.

The concentration response of hOR9Q2 and its mutants to 4-methylphenol was shown in Figure 7. For residue Tyr259, which forms van der Waals interactions with 4-methylphenol, its mutation resulted in a complete loss of function of hOR9Q2, with the response to 4-methylphenol (1000 μM) reduced from 95.69 ± 5.49 to 1.22 ± 0.05. The observed disappearance of the hOR9Q2 response signal can be attributed to the fact that after the mutation at residue Tyr259, the space originally occupied by the large benzene ring of tyrosine and the extensive contact surface it provided disappeared [58]. The methyl group of alanine is utterly incapable of maintaining effective contact with 4-methylphenol, resulting in a drastic reduction in contact area and an increase in the distance between the interacting atomic pairs, thereby causing a sharp weakening of the van der Waals forces. Therefore, the residue Tyr259, as well as the van der Waals forces it forms with the ligand, are critical for hOR9Q2 function.

The mutation of residue Ala108 resulted in a marked reduction in the response signal of hOR9Q2 to 4-methylphenol (1000 μM) from 95.69 ± 5.49 to 33.32 ± 0.98 (Figure 7), indicating that glycine reduces the stability of the *α*-helices in the receptor and alters the solvent-accessible hydrophobic surface area, which affects the hydrophobic interactions between Ala108 and 4-methylphenol. Therefore, it can be concluded that residue Ala108, as well as the hydrophobic interaction force between it and the ligand, contributes to the activation of hOR9Q2 by 4-methylphenol.

Moreover, mutations performed in Cys112, Met207, Phe251, and Leu255 resulted in a complete loss of the response signal of hOR9Q2 to 4-methylphenol (Figure 7). Hydrophobic interaction force and Pi-sulfur bonds were known to play an important role in the binding of proteins to compounds [59,60]. Combined with the fact that the mutation of these four residues results in the absence of hOR9Q2 response signaling, it is hypothesized that changes in the side chains of these four amino acids disrupt the generation of ligand–receptor interaction forces, which in turn disrupts hOR9Q2 function. This is evidenced by the fact that these four amino acid residues (Cys112, Met207, Phe251, and Leu255) are critical components in 4-methylphenol-hOR9Q2 binding, with the hydrophobic interactions and Pi-sulfur bonds established at these positions being of the utmost significance.

Among the four residues identified via sequence alignment, the mutation of Val158 resulted in a complete loss of the response signal of hOR9Q2 in response to 4-methylphenol stimulation (1000 μM). In addition, the mutation of Ile71 was associated with a notable reduction in the response signal, decreasing from 95.69 ± 5.49 to 55.52 ± 3.05 (Figure 7). Studies have shown that alterations in amino acid residues of conserved structural domains attenuate the response signal between the protein and its ligand, thereby having a profound effect on protein function [61,62]. Therefore, it was concluded that these two amino acid residues (Val158 and Ile71) are indispensable for the response of hOR9Q2 to 4-methylphenol. In contrast, the modification of Leu204 resulted in a substantial increase in the response signaling of the receptor, from 95.69 ± 5.49 to 142.67 ± 9.62 (Figure 7). This observation suggests that the mutation of Leu204 might augment the signaling pathway associated with the activation of 4-methylphenol in hOR9Q. Additionally, the alteration of Leu277 did not appear to influence the binding of hOR9Q2 to 4-methylphenol, thus inferring that this amino acid residue is likely not a key factor in the binding interaction.

In the present work, the molecular mechanisms of the interaction between 4-methylphenol and hOR9Q2, as well as the key interaction sites were investigated by computational simulations combined with site-directed mutagenesis. Several limitations should be considered. First, the dose–response relationships for wild-type hOR9Q2 and its mutated amino acids did not reach saturation within the tested concentration range (0–1000 μM), preventing accurate EC_50_ determination. Nevertheless, the complete loss of response across all concentrations for six mutated amino acids provides unambiguous evidence of their essential functional role. Second, the computational simulations parts relied on a single AlphaFold2-predicted structure, and the results may exhibit deviations compared to those based on actual structures. Finally, while this work focused specifically on human OR9Q2, extending this approach to OR9Q2 orthologs from other species would help elucidate the evolutionary conservation and species-specific adaptations of this odorant recognition mechanism.

## 4. Conclusions

In summary, the interaction mechanism between 4-methylphenol and the olfactory receptor hOR9Q2 was studied via computational simulations, and the key binding sites and their contributions were identified in conjunction with site-directed mutagenesis techniques. The results indicated that the cavity formed by transmembrane domains TM3, TM5, and TM6 serves as a pivotal area for the interaction between hOR9Q2 and 4-methylphenol. Through molecular docking techniques, six potential amino acid residues (Ala108, Cys112, Met207, Phe251, Leu255, and Tyr259) in the receptor were identified. Among them, residue Tyr259 was predicted to form van der Waals interactions with 4-methylphenol, with a distance of 2.00 Å. Additionally, residues Ala108, Cys112, Met207, Phe251, and Leu255 formed hydrophobic interactions with the ligand. Specifically, the alkyl groups of Ala108 (3.9 Å), Cys112 (4.3 Å), Met207 (5.2 Å), and Leu255 (4.9 Å) were predicted to form hydrophobic interactions with the carbon atoms of 4-methylphenol, whereas the alkyl groups of Ala108 (4.5 Å), Met207 (4.8 Å), Phe251 (4.6 Å), and Leu255 (5.8 Å) engaged in Pi-alkyl hydrophobic interactions with the aromatic ring of the ligand. Molecular dynamics simulations indicated that the binding conformation of 4-methylphenol with OR9Q2 was stable, with a ΔG_bind_ value of −40.173 ± 0.34 kJ/mol. Cellular experiments confirmed that all six potential amino acid residues identified via molecular docking are essential for the recognition of 4-methylphenol by hOR9Q2, with mutations in five residues (Cys112, Met207, Phe251, Leu255, and Tyr259) resulting in complete loss of function of the receptor, and mutation in one residue (Ala108) leading to partial loss of function. The conserved residue Val158 was also indispensable for hOR9Q2, but this was not the case for Ile71 as the signaling was not lost. The results obtained provide both theoretical insight and data supporting the mechanism of specific activation of hOR9Q2 by 4-methylphenol, as well as a methodological reference for critical site analysis of olfactory receptors. Future research can focus on (1) systematically investigating the perception mechanisms of structurally related phenolic compounds to elucidate the specificity of odorant recognition; (2) comparing the perception mechanisms of phenolic compounds across different species to reveal evolutionary adaptations in odorant recognition.

## Figures and Tables

**Table 1:** Parameters of 4-methylphenol binding to hOR9Q2.

Residues of hOR9Q2	Transmembrane Domains	Distance (Å)	Binding Force	Interaction Category
Ala108	TM3	3.9	Alkyl	Hydrophobic
		4.5	Pi-Alkyl	
Cys112	TM3	4.3	Alkyl	Hydrophobic
		5.0	Pi-sulfur	Miscellaneous
Met207	TM5	4.8	Pi-Alkyl	Hydrophobic
		5.2	Alkyl	
Phe251	TM6	4.6	Pi-Alkyl	Hydrophobic
Leu255	TM6	4.9	Alkyl	Hydrophobic
		5.8	Pi-Alkyl	Hydrophobic

**Table 2:** Binding energy of 4-methylphenol-hOR9Q2 and its constituent parts.

Complex	ΔE_vdW_ (kJ/mol)	ΔE_ele_ (kJ/mol)	ΔG_PB_ (kJ/mol)	ΔG_SA_ (kJ/mol)	−TΔS (kJ/mol)	ΔG_bind_ (kJ/mol)
Protein-Ligand	−81.451 ± 0.351	−4.850 ± 0.091	56.924 ± 0.205	−10.796 ± 0.061	5.699 ± 0.675	−40.173 ± 0.340

Protein-Ligand: The complex of 4-methylphenol and hOR9Q2. ΔE_vdW_: The van der Waals Energy Change. ΔE_ele_: The electrostatic Energy Change. ΔG_PB_: The polar solvation energy change. ΔG_SA_: The non-polar solvation energy change. −TΔS: The contribution of entropy change (ΔS) to the Gibbs free energy change (ΔG) is often not taken into account when comparing binding energies. ΔG_bind_: The binding free energy change, not including −TΔS.

**Table 3:** Mutation information of amino acid residues in hOR9Q2.

No.	Amino Acid Position	Primitive Amino Acid Residues	Original Base Sequence	Mutated Amino Acid Residues	Mutant Base Sequence
1	71	Ile	ATC	Val	GTC
2	108	Ala	GCC	Gly	GGC
3	112	Cys	TGC	Ala	GCC
4	158	Val	GTT	Ile	ATT
5	204	Leu	CTT	Val	GTT
6	207	Met	ATG	Ala	GCG
7	251	Phe	TTC	Ala	GCC
8	255	Leu	CTC	Ala	GCC
9	259	Tyr	TAC	Ala	GCC
10	277	Leu	CTC	Phe	TTC

## Data Availability

The original contributions presented in this study are included in the article/Supplementary Materials. Further inquiries can be directed to the corresponding authors.

## Supplementary Materials

The following supporting information can be downloaded at https://www.mdpi.com/article/10.3390/foods14213738/s1: File S1: Informed Consent Form for Sensory Evaluation; Figure S1: Q-PCR analysis of gene expression after target gene overexpression; Figure S2: Target protein expression after 24 h at different gene concentrations; Figure S3: cAMP activation in response to 4-methylphenol in an OR9Q2-Overexpressing HEK293 cell assay.
