# Supplementary material for: Molecular Mechanism Analysis of the Activation of Human Olfactory Receptor OR9Q2 by 4-Methylphenol
Source: Foods. 2025 Oct 31;14(21):3738. doi: 10.3390/foods14213738 (PMC12609840; doi:10.3390/foods14213738)
Supplement: Supplementary file 1 [file foods-14-03738-s001.zip › Supplementary Material 1-File S1.pdf]

Informed Consent Form for Sensory Evaluation

Research Study Title: Molecular Mechanism Analysis of the Activation of Human Olfactory Receptor OR9Q2 by 4-Methylphenol

Beijing Technology and Business University

No. 33, Fucheng Road, Haidian District

Beijing, 100048, China

April 10, 2023

Description of the Study:

You are invited to participate in a sensory evaluation study conducted by Mengxue Wang. The purpose of this study is to investigate how the compound 4-methylphenol interacts with the human olfactory receptor OR9Q2 and to understand its odor perception mechanisms. This research is funded by the Beijing Life Science Academy Foundation [No. 2023600CA0080], National Natural Science Foundation of China [No. 32302264], Beijing Life Science Academy (BLSA) [No. 2024600CD0310 and No. 2024601QPID08].

Study Procedures:

As a participant in this study, you will be asked to:

1. Attend training sessions to familiarize yourself with odor recognition and ranking procedures.

2. Sniff a series of diluted 4-methylphenol solutions and identify the sample containing the odorant among three options (two blanks and one odorant).

3. Take 5-minute breaks between sample evaluations to avoid olfactory fatigue.

Potential Risks and Discomforts:

1. Olfactory Fatigue: Temporary reduced sensitivity to odors due to repeated exposure.

2. Mild Discomfort: Rare instances of mild nasal irritation.

3. Safety Precautions: All odorants are prepared under controlled laboratory conditions and are safe for inhalation. Participants are not required to ingest any samples.

Confidentiality:

All data collected during this study will remain strictly confidential. Your identity will be anonymized, and results will only be used for research purposes. No personal information will be disclosed in publications.

Voluntary Participation and Right to Withdraw:

Participation in this study is entirely voluntary. You have the right to withdraw your consent and discontinue participation at any time without providing a reason. Withdrawal from the study will not result in any negative consequences.

Contact Information:

For questions or concerns, contact:

Mengxue Wang: sherry_1182@163.com

Consent Statement:

I have read and understood the information provided in this Informed Consent Form. I have had the opportunity to ask questions and received satisfactory answers. By signing below, I voluntarily agree to participate in this study.

Participant's Signature: ______________________________ Date: ____________

Participant's Signature: ______________________________ Date: ____________

Participant's Signature: ______________________________ Date: ____________

Participant's Signature: ______________________________ Date: ____________

Participant's Signature: ______________________________ Date: ____________

Participant's Signature: ______________________________ Date: ____________

Participant's Signature: ______________________________ Date: ____________

Participant's Signature: ______________________________ Date: ____________

Participant's Signature: ______________________________ Date: ____________

Participant's Signature: ______________________________ Date: ____________

Participant's Signature: ______________________________ Date: ____________

Participant's Signature: ______________________________ Date: ____________

Participant's Signature: ______________________________ Date: ____________

Participant's Signature: ______________________________ Date: ____________

Participant's Signature: ______________________________ Date: ____________

Researcher's Signature: ______________________________ Date: ____________

Please print this form for your records.

Thank you for your participation in our study!

Beijing Technology and Business University

Note: This study complies with ethical guidelines approved by the Ethics Committee of Beijing Technology and Business University (Approval No. BTBU202333).
