# Supplementary material for: Molecular Mechanism Analysis of the Activation of Human Olfactory Receptor OR9Q2 by 4-Methylphenol
Source: Foods. 2025 Oct 31;14(21):3738. doi: 10.3390/foods14213738 (PMC12609840; doi:10.3390/foods14213738)
Supplement: Supplementary file 1 [file foods-14-03738-s001.zip › Supplementary Material 2-Figure S1, 2, 3.pdf]

This file includes:

Figure S1. Q-PCR analysis of gene expression after target gene overexpression

Figure S2. Target protein expression after 24 h at different gene concentrations

Figure S3. cAMP activation in response to 4-methylphenol in an OR9Q2-Overexpressing HEK293 cell assay

The concentration of the compound is 0、0.064μM、0.32μM、1.6μM、8μM、40μM、200μM、1000μM; EC50=0.00005333 M; The data are presented as the mean ± SD (n = 3)

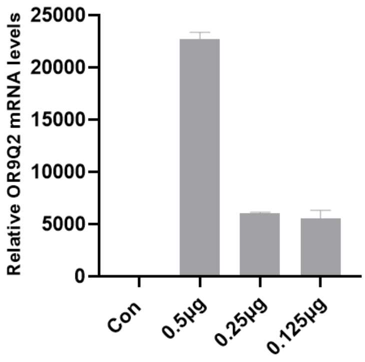

Figure S1. Q-PCR analysis of gene expression after target gene overexpression

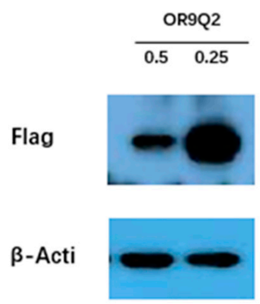

Figure S2. Target protein expression after 24 h at different gene concentrations

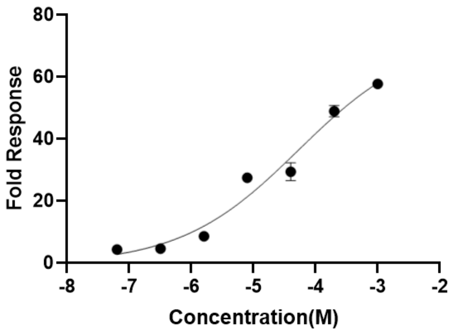

Figure S3. cAMP activation in response to 4-methylphenol in an OR9Q2-Overexpressing HEK293 cell assay

The concentration of the compound is 0、0.064μM、0.32μM、1.6μM、8μM、40μM、200μM、1000μM; EC50=0.00005333 M; The data are presented as the mean ± SD (n = 3)
